# A comparative analysis of depressive-like behavior: Exploring sex-related differences and insights

**DOI:** 10.1371/journal.pone.0294904

**Published:** 2023-11-29

**Authors:** Ana Carolina Cavalcante Rodrigues, Caroline Vitória de Lima Moreira, Camila Carlos Prado, Luan Silvestro Bianchini Silva, Rafael Fernandes Costa, Adesina Paul Arikawe, Gustavo Rodrigues Pedrino, Elson Alves Costa, Osmar Nascimento Silva, Hamilton Barbosa Napolitano, Iranse Oliveira-Silva, James Oluwagbamigbe Fajemiroye

**Affiliations:** 1 Instituto de Ciências Biológicas, Universidade Federal de Goiás, Goiânia, Goiás, Brazil; 2 Graduate Program in Pharmaceutical Sciences, Campus Arthur Wesley Archibald, Evangelical University of Goiás, Anápolis, Brazil; 3 Department of Physiology, Faculty of Basic Medical Sciences, College of Medicine, University of Lagos, Idi-Araba, Lagos, Nigeria; 4 Grupo de Química Teórica e Estrutural de Anápolis, Universidade Estadual de Goiás, Anápolis, Goiás, Brazil; University of Lübeck: Universitat zu Lubeck, GERMANY

## Abstract

Profiling the variability related to the estrous cycle is essential for assessing depressive-like behavior and screening drugs. This study compares circulating plasma corticosterone levels [CORT] and behavioral alterations in mice exposed to sucrose preference, forced swimming, and tail suspension tests (SPT, FST, and TST, respectively). While SPT exposure did not significantly alter [CORT], FST and TST showed notable changes. Mice in the TST exhibited increased movement and decreased immobility time compared to FST, suggesting a lower likelihood of depressive-like behavior in male mice. Notably, during the proestrus phase, female mice displayed the highest tendency for depressive-like behavior and elevated [CORT], but similar response to antidepressants (imipramine and fluoxetine). The inherent stress of the FST and TST tasks appears to influence [CORT] as well as depressant and antidepressant effects. These comparisons provide valuable insights for further behavioral phenotyping, model sensitivity assessment, and deepen our neurobiological understanding of depression in the context of drug screening.

## Introduction

Behavioral, biochemical, and sex-related profiling of animals under stressful situations is a prerequisite to understanding the pathophysiology of depression and effective drug screening [1, 2]. Behavioral parameters from the sucrose preference test (SPT), forced swimming test (FST), and tail suspension test (TST) are being used to establish depressive or antidepressant-like behaviors in rodents [3–5]. Stressful situations often activate the hypothalamic-pituitary-adrenal (HPA) axis to release corticotropin-releasing hormone (CRH) from the hypothalamic paraventricular nucleus [6]. CRH stimulates the release of the adrenocorticotrophic hormone, which precedes the release of cortisol in humans and corticosterone in rodents [7]. Stressful exposure to novel environments or tasks could increase the circulating level of these hormones.

The FST and TST are widely accepted models of depression developed in 1977 and 1985 by Porsolt and Steru, respectively [3, 8]. When a rodent is exposed to an inescapable stressful task in the FST, the animal will adopt an immobile posture after an initial attempt to escape [3]. Similarly, the animal submitted to the TST often exhibits an escape behavior, such as climbing, before reaching a state of immobility [8]. Treatment with antidepressants can reduce the immobility time and increase movement or swimming time. In this context, the response to fluoxetine (a selective serotonin reuptake inhibitor) and imipramine (a tricyclic antidepressant) treatment could provide data on the predictive validity of these models. In the SPT model, rodents are given a free choice between two separate bottles, one filled with water and the other with a 1% sucrose solution. Anhedonia, one of the symptoms of depression, is assessed in this model through mice consumption preferences [4].

Despite the usefulness of the SPT, FST, and TST in studies on depression and drug screening, the stress-related depressive-like behavior and hormonal changes in animals exposed to these models remain poorly explored and assessed. Similarly, the influence of sex on these changes and how this relates to response at the proestrus and estrus phases of the estrous cycle is not well understood. This is against the backdrop that some parameters that are relevant to animal behavior, physiology, and pharmacology can oscillate at different stages of the estrous cycle [9–13]. Thus, the present study evaluates and compares alterations in behavioral parameters and corticosterone levels of female mice (at proestrus or estrus phases) and male mice exposed to SPT, FST, and TST.

## Material and methods

### Experimental animals

Swiss albino female mice (without estrous cycle identification, at proestrus and estrus phases) and male mice weighing between 30 ± 2 g (6 weeks old) were used in this study. Animals were procured from the animal center, Federal University of Goiás (UFG), and kept under controlled conditions of temperature (22°C) and lighting (12-hour light/dark cycle with lights on at 07:00h), with access to food and water *ad libitum*. Animals were allowed to acclimatize for seven days before the commencement of the experiments under an illumination level of approximately 40 lux. The experiment was conducted between 1200 and 1800 hours in compliance with the approved experimental protocol. After the behavioral evaluations, animals subjected to cardiac puncture following intraperitoneal anesthesia with 300 mg/kg tribromoethanol to collect blood samples for corticosterone quantification using the high-performance liquid chromatography (HPLC) method. The corticosterone baseline was measured in the sham group of animals without exposure to behavioral tests. All experimental protocols complied with the International Principles for Biomedical Research involving animals and were carried out after approval from the Ethics Committee on the Use of Animals (CEUA/ Nº. 063/19) of UFG. All efforts were made to minimize the suffering of the animals.

Scheme 1 illustrates the experimental timeline for randomly allocated male and female mice after seven days of acclimatization. The SPT, FST, and TST were conducted using independent mice in each experimental setup to avoid potential test battery effects. Following the completion of each test, blood samples were collected immediately. All the behavioral test sessions were videotaped, and the behavioral parameters were subsequently scored and analyzed by experienced, independent, and blind observers. These blood samples were collected for quantifying corticosterone levels using HPLC analysis.




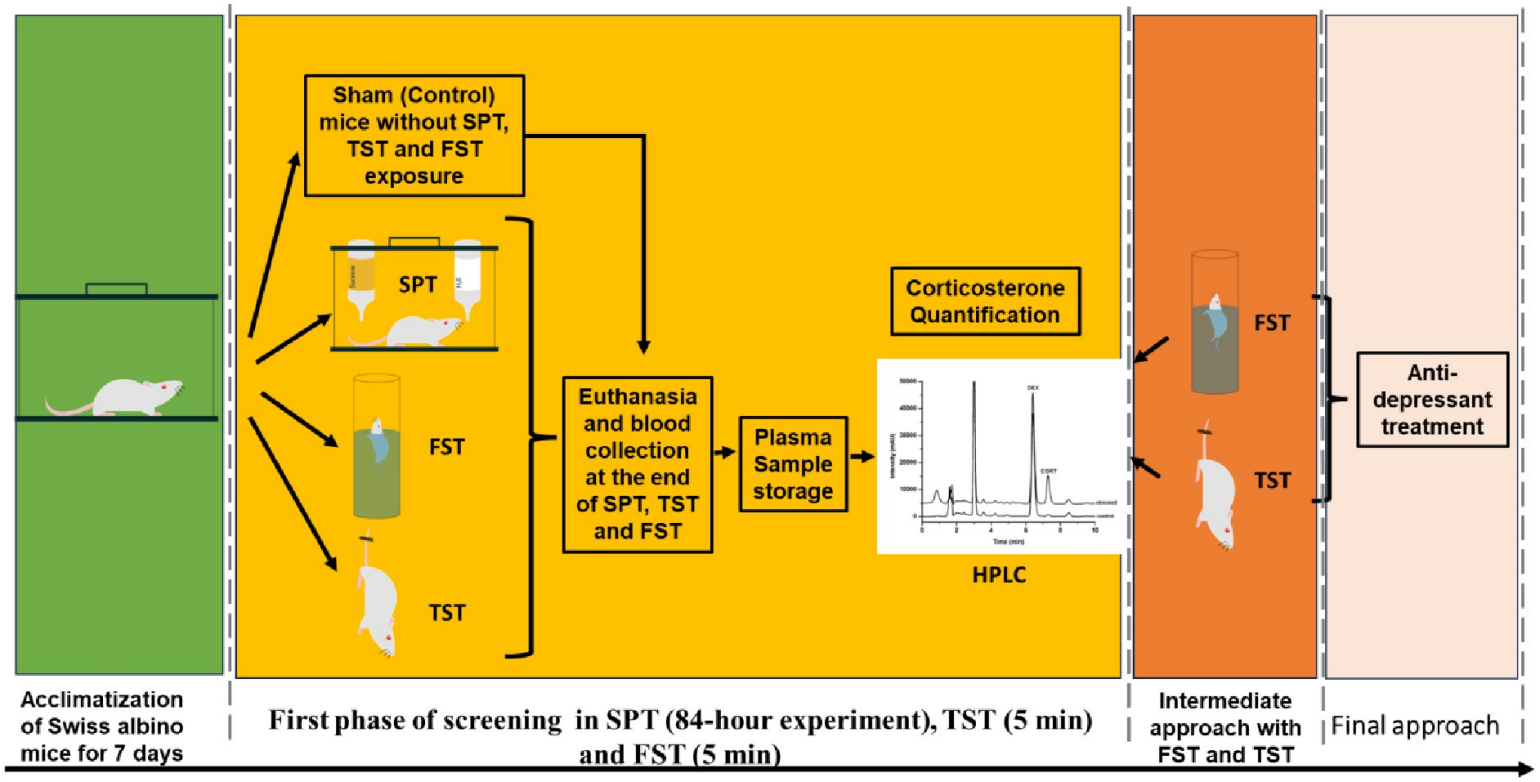




**Scheme 1. Experimental timeline.** The screenings were carried out in three different phases: i. First phase, ii. Intermediate phase, and iii. Final approach. At the first phase, mice were randomly separated into group of sham (control) mice without sucrose preference test (SPT), and experimental group with SPT, forced swimming test (FST) or tail suspension test (TST) exposure after 7 days of acclimatization. The intermediate and final approach focused on the estrous cycle (proestrus and estrus) and antidepressant treatment related response of mice exposed to FST or TST.

### Estrous cycle assessment

Vaginal cytology permits the evaluation of the estrous cycle in female mice. To collect cells from the vaginal cavity, a cotton-tipped vaginal swab wetted with 1 ml of saline (0.9%) was gently inserted into the vagina. These cells were then analyzed microscopically, as previously described [14]. The cells on the swab were transferred onto a glass slide, and images of the cells were captured using a light microscope with a 10x objective lens. The estrous cycle typically consists of four to five phases: proestrus, estrus, diestrus I, and diestrus II, with an average duration of four to five days [14–18]. Each phase is characterized by different types of predominant cells [15]. Large, globose, and nucleated cells are predominant at the proestrus phase. The estrus phase is characterized by cornified and keratinized cells, while leukocytes or a mixture of globose and nucleated cells are the main cell types at diestrus phases I and II [15, 19].

### Behavioral testing procedures

#### Sucrose preference test (SPT)

Rodents naturally prefer sweet drink solutions (sucrose solution) to water in a two-bottle free-choice regimen [20]. SPT is commonly used to assess stress-induced anhedonia and involves providing rodents with a choice between two separate bottles: one containing water and the other containing a 1% sucrose solution. Previous studies have suggested that a sucrose concentration of 1–2% (wt/vol) is optimal for detecting depressive-like behavior in mice or rats [20–23]. Since sucrose tends to produce hedonic behavior in rodents, the analysis of sucrose preference becomes relevant [4]. Typically, two bottles are available in the cages, one with water and the other with sucrose solution. The experimental protocol involves a habituation phase, followed by basal and preference test consumption measurements. During the habituation phase, mice are exposed to 48 hours of access to two bottles: one containing 1% (wt/vol) sucrose solution and the other containing tap water, with free access to lab pellet chow. Subsequently, mice are moved to the experimental room for the test session, where basal consumption is measured over 12 hours and preference test consumption is measured over 24 hours. The weight of the mice and the volume of liquid in the bottles are measured. The sucrose preference percentage (%) is calculated as follows: sucrose solution consumption (g) / (sucrose solution consumption [g] + water consumption [g]) × 100%.

#### Forced swimming test (FST)

The FST permits the study of depressive behaviors in animals. In this model, the animal is placed and kept floating for five minutes in a cylindrical apparatus filled with water (height: 42 cm; diameter: 18 cm; containing 30 cm of water at 25 ± 1°C), maintained at room temperature of approximately 26 ± 1°C. Initially, the mice display active attempts to escape through vigorous activity and active swimming before assuming a posture of immobility [5]. In this study, active swimming or mobile attempts were measured indicators of movement, while a mouse floating motionless and making minimal movements to keep its head above water was considered immobile [24]. The duration of movement and immobility was recorded during the testing period. The experimental plastic cylinders were cleaned with a 10% ethanol solution before the test. The test session was videotaped, and the behavioral parameters were later scored and analyzed by experienced observers who were independent and blind to the experimental conditions. In a separate experiment involving pharmacological intervention, mice were treated with fluoxetine (FLX 15 mg/kg, p.o.), imipramine (IMI 15 mg/kg, p.o.), or vehicle (A mixture of 0.9% NaCl and 5% (v/v) Tween-80 at 10 mL/kg p.o.) sixty minutes before the FST. The dose of the reference drugs was determined through pilot experiments and published work [5].

#### Tail suspension test (TST)

The TST proposed by Steru in 1985 [8] is a widely used animal model for studying depression. In this test, the animal is suspended 50 cm above the floor by the tail using adhesive tape placed approximately 1 cm from the tip of the tail. The duration of immobility and movement is recorded for five minutes [8, 25]. Immobility time is measured when mice hang passively or remain motionless. Before each test, the experimental bench is cleaned with a 10% ethanol solution. This test session is videotaped, and the behavioral parameters are later scored and analyzed by experienced observers who are independent and blind to the experimental conditions. In a separate experiment involving pharmacological intervention, mice were treated with fluoxetine (15 mg/kg, p.o.), imipramine (15 mg/kg, p.o.), or vehicle (10 mL/kg p.o.) sixty minutes before the TST.

### Biochemical analysis

#### Quantification of corticosterone

After the behavioral testing procedures, plasma samples were collected by centrifuging whole blood samples at 10,000 g for 10 minutes. According to the previous study [26], the plasma samples were stored at -20°C until further analysis using high-performance liquid chromatography (HPLC). Briefly, dichloromethane (100 μL) was added to each plasma sample (100 μL) and vortexed for 30 seconds. The mixture was then centrifuged for 5 minutes at 10,000 rpm, and the organic layer was carefully removed and dried. The residue was reconstituted in 1 mL of the mobile phase, which consisted of a mixture of 0.1% acetic acid and acetonitrile (65:35, v/v). Finally, 50 μL of the reconstituted sample was injected into the HPLC system. Before use, the mobile phase was degassed in an ultrasonic bath (Unique® USC-2800) for 20 minutes. Biochemical analysis was performed using a Shimadzu HPLC system (Shimadzu, Kyoto, Japan) equipped with an LC-20AT pump, DGU-20A5 degasser, SPD-M20A PDA detector, CTO-20A column oven, and SIL-20A autosampler. The column oven temperature was set at 30°C, and a Shimadzu® C18 column (250 × 4.6 mm, 5 μm) was used for separation. The flow rate was set at 1.0 mL/min, and detection was performed at a specific wavelength of 245 nm. The data were processed using LabSolutions software. The quantification procedure was adapted from previously described methods [26, 27].

### Statistical analysis

The statistical analysis and graphical representations of data were performed using the software GraphPad Prism v8. Data were expressed as mean ± standard error of the mean (SEM) with a sample size of 10 animals per group. Student’s t-test was used to compare the variables between two groups, while one-way analysis of variance (ANOVA) was used for analyzing data with more than two groups. Two-way ANOVA was used to evaluate sex vs. [CORT] or drug intervention prior to Bonferroni test as a post hoc test. Pairwise follow-up comparisons of individual treatment groups were carried out using the Bonferroni test as a post hoc test. Parametric testing was carried out after assessing the normality of the experimental data with the Shapiro–Wilk test. Statistical significance was defined as p < 0.05.

## Results

### Comparison of SPT, FST, and TST using corticosterone levels

**Fig 1:**
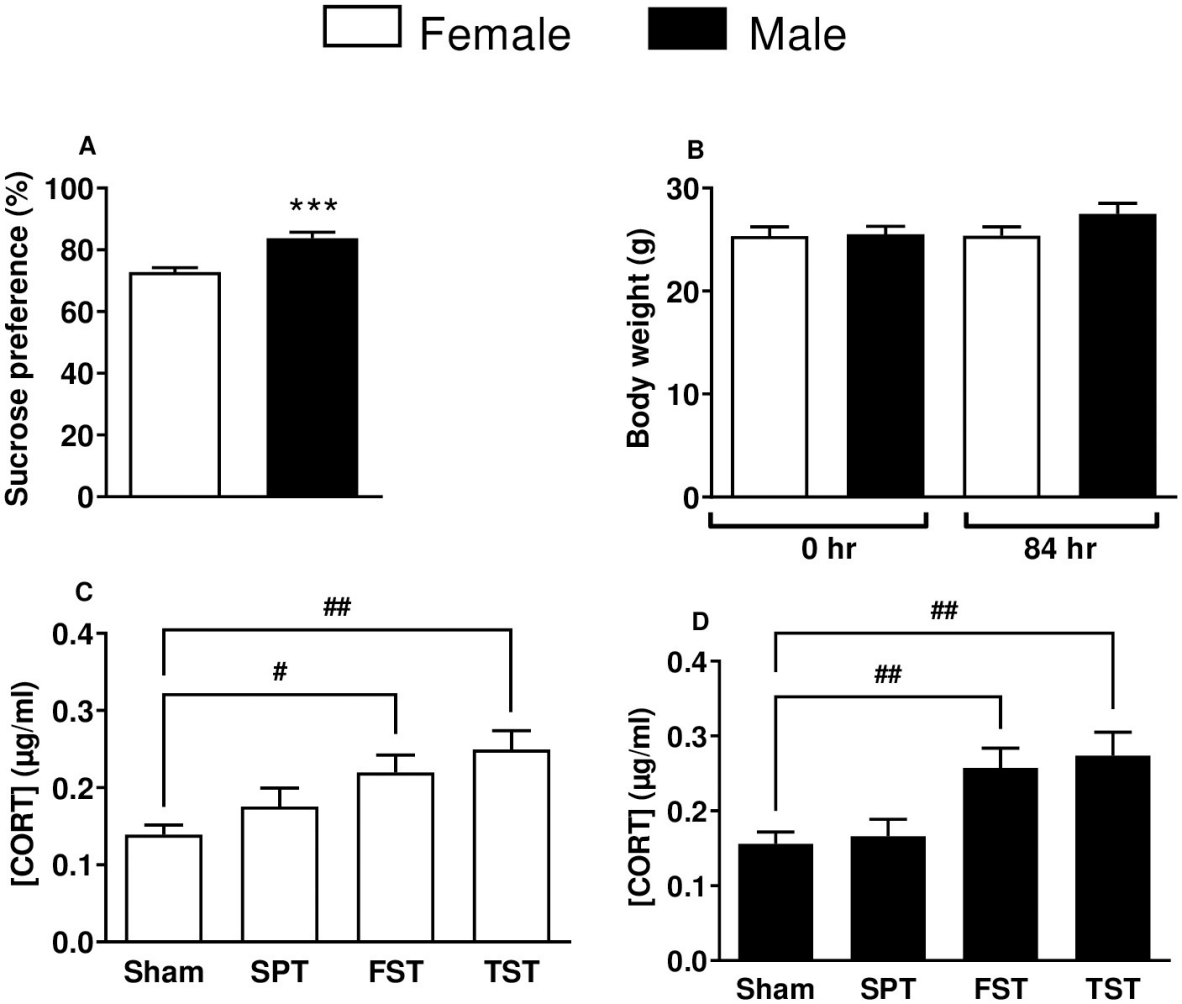
Behavioral changes in adult Swiss mice exposed to sucrose preference (SPT), forced swimming (FST), and tail suspension (TST) tests. Males (n = 10) and female mice (n = 10, 5 at the proestrus phase + 5 at the estrus phase of the estrous cycle) were used. A) Weight (g) at 0 hours and at the end of the 84-hour experiment (48 hours of habituation, 12 hours of baseline, and 24 hours of SPT session). (B) Blood plasma corticosterone levels [CORT] (μg/mL) in groups of female (C) and male (D) mice without (Sham) and with exposure to SPT, FST, and TST. The bars represent sucrose preference in percentage (%). The white and black bars represent female and male mice, respectively. Data are presented as mean ± SEM. *** p < 0.001 compared to female mice with sucrose consumption, while # p < 0.05 and ## p < 0.001 compared to sham mice without SPT, FST, and TST exposure (n = 10).

The Fig 1A showed a higher (p < 0.001) sucrose preference in male (83.7 ± 2.0%) than female (72.8 ± 1.4%) mice without recording significant weight change (Fig 1B). The Fig 1C and 1D showed different corticosterone levels (μg/mL) in the plasma samples of female and male mice, following SPT, FST or TST exposure. A significantly higher (p < 0.05) levels of corticosterone were measured in the female group [Fig 1C; FST (0.22 ± 0.07 μg/mL and TST (0.25 ± 0.08 μg/mL), n = 10] as well as in the male group [Fig 1D; FST (0.26 ± 0.03 μg/mL) and TST (0.27 ± 0.03 μg/mL), n = 10] compared to their respective control groups (sham mice). However, both female and male mice exposed to SPT did not show significant difference in the levels of corticosterone as compared to their respective control groups.

### FST- and TST-oriented comparison using behavioral parameters

**Fig 2:**
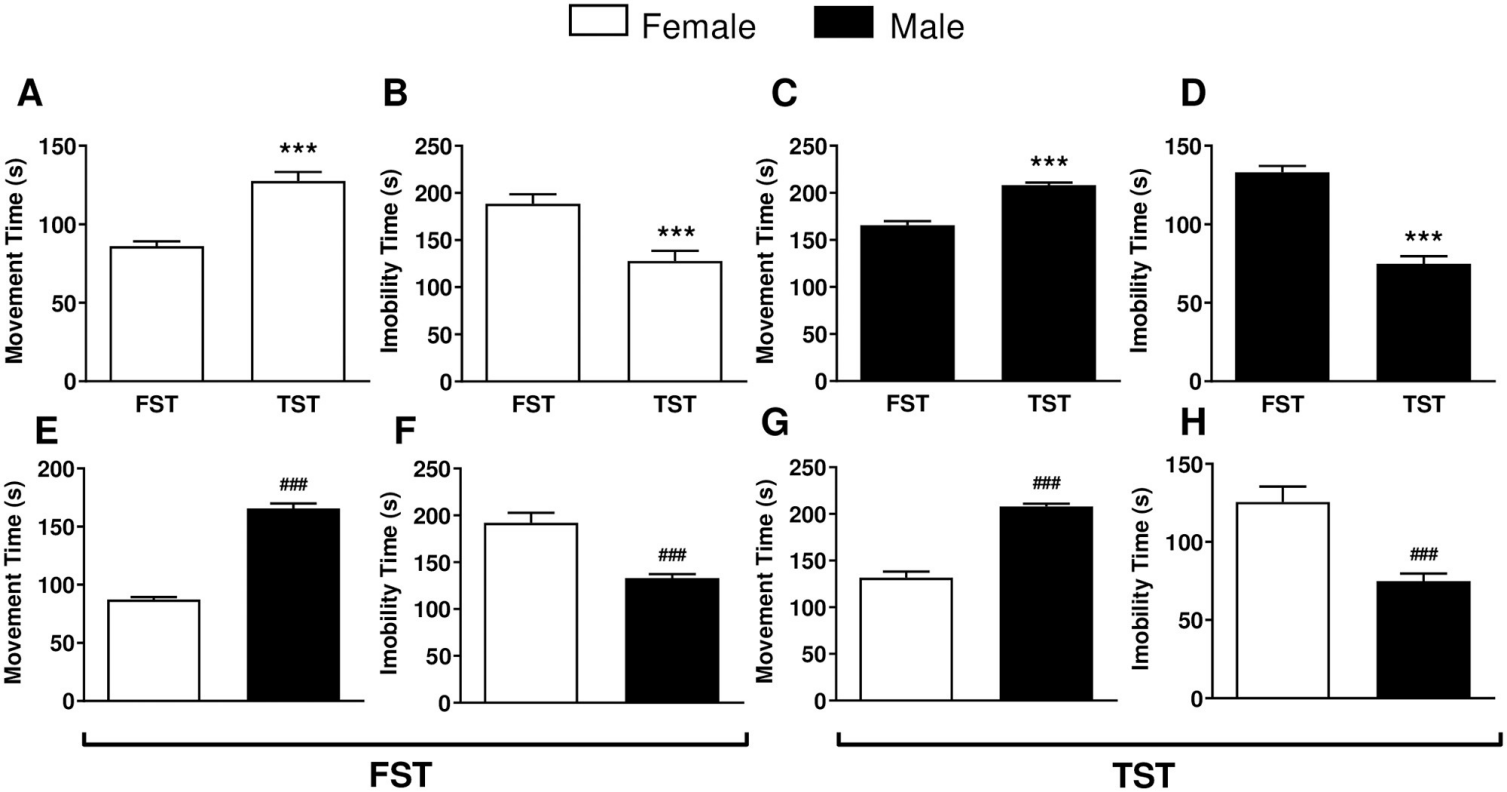
Comparative analysis of forced swim (FST) and tail suspension (TST) tests in male in adult Swiss mice. (A) Movement time (s) and (B) immobility time (s) in female mice (n = 10, 5 mice at the proestrus phase + 5 mice at the estrus phase of the estrous cycle) in FST; (C) Movement time (s) and (D) immobility time (s) in male mice (n = 10) in FST; (E) and (F) in FST compare behavioral parameters (movement and immobility time) between female and male mice; (G) and (H) in TST compare behavioral parameters (movement and immobility time) between female and male mice. The white and black bars represent female and male mice, respectively. Data are presented as mean ± SEM. *** p < 0.001 compared to behavioral parameters in FST, while ### p < 0.001 compared to the female mice group exposed to FST or TST (n = 10).

Fig 2A shows a significant difference (p < 0.001) between mean movement time in female mice exposed to FST (86.1 ± 3.1 s) and TST (127.6 ± 5.7 s). The mean immobility time was also significantly different (p < 0.001) in female mice exposed to FST (Fig 2B; 188.5 ± 10.1 s) and TST (Fig 2B; 127.8 ± 10.8 s). In the male experimental group, the time of movement in the FST (Fig 2C; 165.6 ± 4.3 s) was significantly lower (p < 0.001) as compared to the one recorded in the TST (Fig 2C; 208.0 ± 2.9 s). The immobility time in the male mice under FST (Fig 2D; 133.0 ± 4.2 s) was significantly different (p < 0.001) as compared to the immobility time under TST (Fig 2D; 74.8 ± 4.9 s) in the male mice. Fig 2 also shows the significant difference (p < 0.001) between groups of male and female mice subjected to FST (Fig 2E and 2F) and TST (Fig 2G and 2H) using movement and immobility times as behavioral parameters.

### Comparison of behavioral parameters in male mice and female mice at proestrus and estrus phases

**Fig 3:**
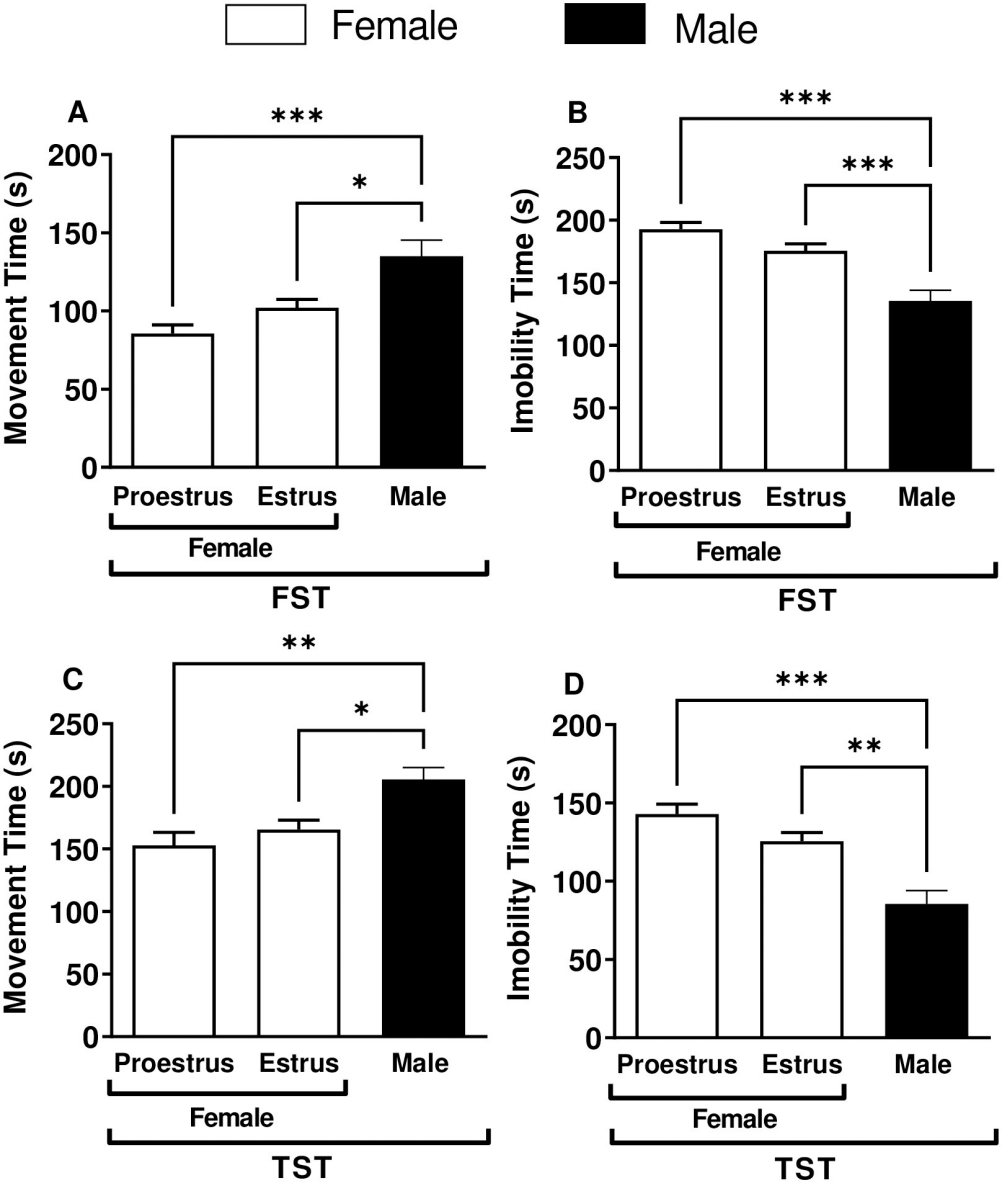
Comparison of behavioral alterations in female at the proestrus and estrus phases of the estrous cycle vs. male swiss mice exposed to forced swim test (FST) and tail suspension test (TST). (A) Movement time (s) in FST for male and female mice at the proestrus and estrus phases. (B) Immobility time (s) in FST for male and female mice at the proestrus and estrus phases. (C) Movement time (s) in TST for male and female mice at the proestrus and estrus phases. (D) Immobility time (s) in TST for male and female mice at the proestrus and estrus phases. The white and black bars represent female and male mice, respectively. Data are presented as mean ± SEM. * p < 0.05, ** p < 0.01, and *** p < 0.001 compared to female mice at the proestrus and estrus phases groups (n = 10).

Fig 3 shows the behavioral difference between groups of male and female mice at the proestrus, and estrus phase subjected to FST (Fig 3A and 3B) and TST (Fig 3C and 3D). The mean movement time between female mice at the proestrus phase (85.6 ± 5.5 s) was not significantly different from that of female mice at the estrus phase (102.0 ± 5.3 s; Fig 3A) after FST exposure. Likewise, the average immobility time in the female mice at the proestrus phase (192.0 ± 5.3 s) was also not significantly different from female mice at the estrus phase (175.5 ± 5.5 s; Fig 3B) in this model. In Fig 3C for TST, the mean movement time of the female mice at the proestrus phase (152.6 ± 10.3 s) was not significantly different from that of the female mice at the estrus phase (165.5 ± 7.5 s; Fig 3C) as the immobility time in the female mice at the proestrus phase (142.8 ± 6.4 s) was also not different from that of the female mice at the estrus phase (125.1 ± 5.5 s; Fig 3D). However, both movement and immobility times were significantly increased and reduced, respectively, in male mice as compared to female mice at proestrus and estrus phases subjected to FST and TST (p < 0.05).

### Comparison of corticosterone levels in male mice and female mice at proestrus and estrus phases

**Fig 4:**
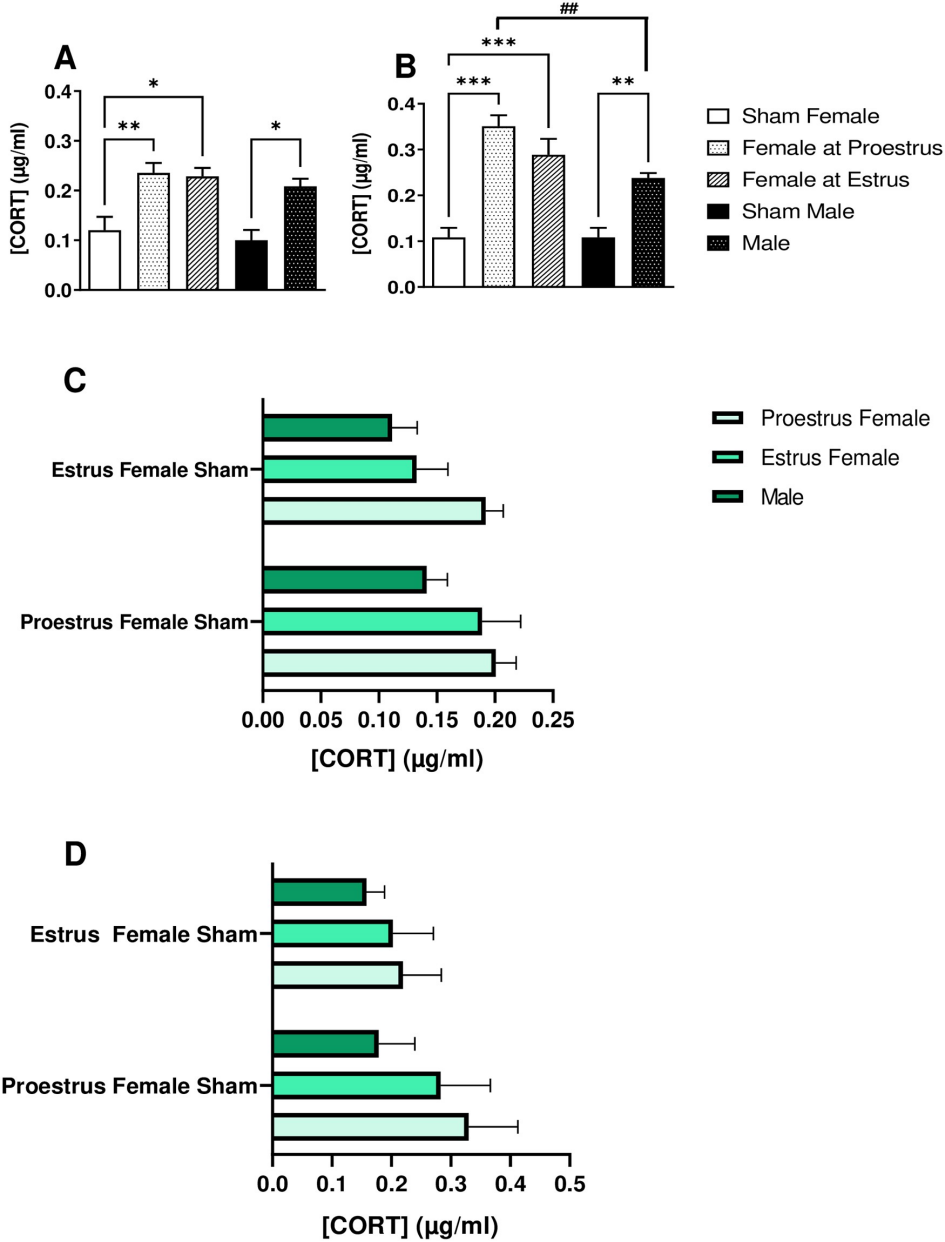
Comparison of blood plasma corticosterone levels in male and female Swiss mice exposed to forced swim test (FST) and tail suspension test (TST). (A) forced swim (FST) and (B) tail suspension (TST) tests. Data are presented as mean ± SEM; * p < 0.05, ** p < 0.01, and *** p < 0.001 compared to sham group of female (proestrus and estrus) or male mice (n = 5), while ## p < 0.01 compared to female mice at proestrus in FST or TST (n = 5). Two independent variables [i. sex/ hormonal background, and ii. experimental intervention—sham female at proestrus or estrus and behavioral tests—FST (C) or TST (D)] with [CORT] representing a dependent variable were subjected to a Two-way ANOVA prior to Bonferroni test as a post hoc test.

The level of corticosterone in male mice after FST (Fig 4A; 0.21 ± 0.01 μg/ml) was not different significantly from that of the female mice at proestrus (Fig 4A; 0.24 ± 0.02 μg/ml) and estrus (Fig 4A; 0.23 ± 0.02 μg/ml) phase. However, the data from TST showed a significant difference in corticosterone levels of male mice (Fig 4B; 0.24 ± 0.01 μg/ml) as compared to female mice at proestrus (Fig 4B; 0.35 ± 0.02 μg/ml). Although the corticosterone levels at proestrus (FST 0.24 ± 0.02 μg/ml and TST 0.35 ± 0.02 μg/ml, Fig 4A and 4B, respectively) and estrus (FST 0.23 ± 0.02 μg/ml; TST 0.29 ± 0.03 μg/ml, Fig 4A and 4B, respectively) phase of animals exposed to FST and TST were significantly increased in comparison with the sham groups of female (FST 0.12 ± 0.03 μg/ml and TST 0.11 ± 0.02 μg/ml) and male mice (FST 0.10 ± 0,02 μg/ml and TST 0.11 ± 0.02 μg/ml), no significant different was recorded between animals at proestrus and estrus phase of estrous cycle after behavioral tests. The corticosterone levels in male mice exposed to FST and TST were significantly increased in comparison with the sham groups of female or male mice. In the FST, statistical analysis showed no sex–[CORT] related interaction (F (1, 16) = 9.6 x 10–32, p > 0.05, Fig 4A, two-way ANOVA prior to Bonferroni test as a post hoc test). Although the sex–[CORT] interaction was not statistically different [F (1, 16) = 1.93, p = 0.18] in the TST, the row factor (female and male) was significantly different [F (1, 16) = 13.15, p = 0.002, Fig 4B, two-way ANOVA prior to Bonferroni test as a post hoc test].

The estrous cycle stage-dependent [CORT] was further explored in a separate experiment (Fig 4C and 4D) using two-way ANOVA of two independent variables (sex/hormonal background and experimental intervention) prior to the Bonferroni test as a post hoc test. Unlike column factor [proestrus female, estrus female, and male; F (2, 24) = 4.63, p = 0.592], the p-value for interaction [F (2, 24) = 0.54, p = 0.002] and row factor [proestrus female sham and estrus female sham; F (1, 24) = 2.83, p = 0.105] was not significantly different in the FST (Fig 4C). However, in the TST, the p-value for column factor [proestrus female, estrus female, and male; F (2, 24) = 6.53, p = 0.005] and row factor [proestrus female sham and estrus female sham; F (1, 24) = 8.16, p = 0.009] was significantly different without interaction [F (2, 24) = 1.16, p = 0.331] between row and column factor.

### Comparison of behavioral alterations in mice treated with antidepressants

**Fig 5:**
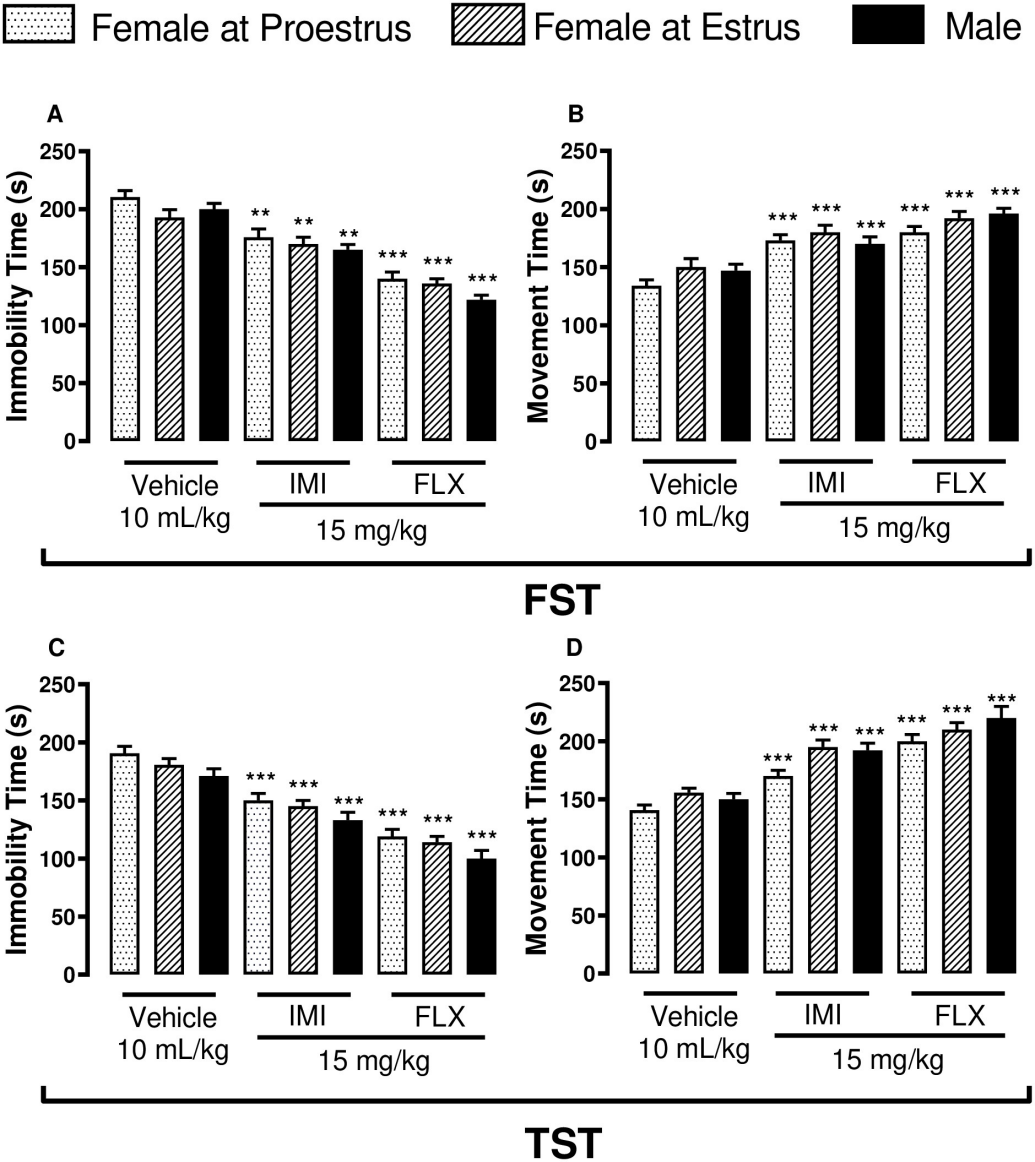
Effects of antidepressant treatment on immobility and movement time of Swiss mice exposed to the forced swimming test (FST) and tail suspension test (TST). Oral treatment with either imipramine or fluoxetine at a dose of 15 mg/kg on immobility (A) and movement time (B) in FST; as well as immobility (C) and movement time (D) in TST. Data are presented as mean ± SEM. ** p < 0.01 and *** p < 0.001 (n = 10) compared to vehicle-treated groups of female mice at the proestrus phase (n = 10).

Comparisons of data in the FST with; i. average immobility time of females at proestrus with vehicle (210.20 ± 5.32), IMI (176.00 ± 7.02) or FLX (140.00 ± 6.00) treatment showed significant difference [F (2, 27) = 32.53, Fig 5A]; females at estrus with vehicle (193.30 ± 6.73), IMI (170.00 ± 6.04) or FLX (136.10 ± 4.06) treatment showed significant difference [F (2, 27) = 25.46, Fig 5A; and males with vehicle (200.00 ± 5.02), IMI (165.00 ± 4.63) or FLX (122.00 ± 4.00) treatment showed significant difference [F (2, 27) = 73.66, Fig 5A; ii. Average movement time of females at proestrus with vehicle (134.00 ± 5.00), IMI (173.00 ± 5.02) or FLX (180.00 ± 5.01) treatment showed significant difference [F (2, 27) = 24.57, Fig 5B]; females at estrus with vehicle (150.00 ± 7.41), IMI (180.00 ± 6.04) or FLX (192.00 ± 6.01) treatment showed significant difference [F (2, 27) = 11.10, Fig 5B; and males with vehicle (147.20 ± 5.45), IMI (170.00 ± 6.04) or FLX (196.00 ± 4.53) treatment showed significant difference [F (2, 27) = 20.92, Fig 5B; while in the TST with i. immobility of females at proestrus with vehicle (190.10 ± 6.00), IMI (150.00 ± 6.07) or FLX (119.00 ± 6.06) treatment showed significant difference [F (2, 27) = 35.81, Fig 5C]; females at estrus with vehicle (180.10 ± 5.55), IMI (145.00 ± 5.02) or FLX (114.00 ± 5.08) treatment showed significant difference [F (2, 27) = 41.69, Fig 5C; and males with vehicle (171.00 ± 6.28), IMI (133.00 ± 6.77) or FLX (100.00 ± 7.07) treatment showed significant difference [F (2, 27) = 27.86, Fig 5C; ii movement of females at proestrus with vehicle (140.60 ± 4.33), IMI (170.00 ± 5.05) or FLX (200.00 ± 5.61) treatment showed significant difference [F (2, 27) = 34.16, Fig 5D]; females at estrus with vehicle (155.00 ± 4.00), IMI (195.00 ± 6.00) or FLX (210.00 ± 6.05) treatment showed significant difference [F (2, 27) = 26.91, Fig 5D; and males with vehicle (150.00 ± 5.09), IMI (192.00 ± 6.01) or FLX (220.00 ± 10.09) treatment showed significant difference [F (2, 27) = 22.61, Fig 5D]. In the FST, statistical analysis did not show interaction [F (4, 81) = 0.99, p = 0.41, two-way ANOVA prior to Bonferroni test as a post hoc test] between the sex or hormonal background (column factors)—female at proestrus, female at estrus and male and pharmacological intervention/ treatments (row factors)—Vehicle, IMI and FLX using immobility parameter (dependent variable) in Fig 5A. With the movement parameter in Fig 5B of FST, the interaction was not significant for both column and row factors [F (4, 81) = 0.91, p = 0.46, two-way ANOVA prior to Bonferroni test as a post hoc test]. A significant difference (p<0.05) was detected for both the column and row factors using immobility [F (2, 81) = 4.52 and F (2, 81) = 116.10, respectively] and movement [F (2, 81) = 3.44 and F (2, 81) = 50.86, respectively] time parameters. In the TST, the interaction between the column and row factors was not significant using immobility time [F (4, 81) = 0.07, p = 0.99, two-way ANOVA prior to Bonferroni test as a post hoc test, Fig 5C] and movement time [F (4, 81) = 0.79 p = 0.53, two-way ANOVA prior to Bonferroni test as a post hoc test, Fig 5D] as dependent variables. A significant difference (p<0.05) was detected for both the column and row factors using immobility time [F (2, 81) = 7.35 and F (2, 81) = 101.60, respectively, Fig 5C] and movement time [F (2, 81) = 7.79 and F (2, 81) = 77.82, respectively, Fig 5D] parameters.

## Discussion

Preclinical *in vivo* studies of disease and drug candidates with therapeutic potential are prerequisites to clinical trials [28]. Hence, comparative consideration for sex or model-related variables is imperative in complex depressive-like behaviors. SPT, FST, and TST are widely used to study depression and screen antidepressant drugs.

In the SPT, the habituation of mice and baseline measurement of sucrose ingestion are fundamental to depressive-like behavior assessment [4]. In this study, in comparison with male mice, the female mice ingested a relatively lower percentage of sucrose solution without weight change. As the sucrose preference measures a core symptom of depression–anhedonia [2, 29], lower sucrose ingestion suggests a higher tendency of depressive-like behavior and a sex-linked variable.

As the SPT, FST, and TST are associated with different tasks and stress levels, the analysis of the corticosterone levels of mice was used as a comparative parameter in each of these behavioral paradigms. Stress researchers have focused on corticosterone release for decades as it impacts male and female animal behavior [28, 29]. The analysis of plasma samples from mice exposed to FST and TST in this study showed an increase in corticosterone levels. Since there was no significant change in the level of this hormone in animals that were exposed to SPT, hypothetically, mice performance in this test seems to be passive with the lowest chance of stressful task. The unpredictable forced swim and tail suspension tasks often exposed mice to an unpleasant and unfamiliar environment [30] that demands survival and escape-oriented strategy. Unlike the changing interest or passive ingestive preference in SPT, the repeated active struggle in FST and TST suggests behavioral readout of stressful situations aligns with high plasma corticosterone levels. Although corticosterone level was lower in animals after FST exposure than TST exposure, this finding supports additional comparative assessment of both models (FST and TST) to gain insight into model and sex-related changes.

A comparative difference in the behavior of mice exposed to FST and TST was conducted using parameters such as movement and immobility time. Irrespective of sex, significantly lower and higher mean time of movement and immobility, respectively, in mice exposed to FST as compared to mice exposed to TST suggest model-related variable capable of impacting overall behavioral endpoint. The lack of a clear-cut readout between immobile posture and movement behavior is associated with timing discrepancy in these models. The temporal oscillatory patterns in immobility and movement behavior may suggest indecision, emotional instinct, or intrinsic biological variables to be explored in future investigations. Additional analysis and comparison reveal sex differences in behavioral patterns. Sex differences in the prevalence, symptomatology, and treatment response among psychiatric patients support sex-oriented behavioral investigation in animal models of depression [30]. Depressive males and females exhibit differences in clinical presentation, course, and response to antidepressant intervention [31]. The analysis of the changes in movement and immobility time between a group of female and male mice which showed statistically significant results supports sex-dependent behavioral changes. A previous study in female rats showed a longer pretest swimming time and floating duration with less climbing [32]. Meanwhile, comparative analysis of behavioral changes at the proestrus or estrus phases remains poorly explored. The proestrus phase prepares the animal for the heat phase (estrus) before mating and conception. At these phases, mice are likely to be receptive to mating when paired with sexually mature males. A comparative study of mice at these phases is fundamental to our subsequent research program on peripartum depressive-like behavioral profiling in TST and FST. In female rats, active behaviors, swimming, and climbing increased at the proestrus or estrus phases [33]. These behavioral changes were not significant from those of rats at the metestrus/diestrus phases. A separate study that reported higher immobility time in female rats at the proestrus or estrus phase as compared to the male rats deepens the scope of behavioral variability and calls for careful evaluations to ensure consistent findings. The present study demonstrated a higher movement time and lower immobility time of male mice exposed to the FST and TST as compared to female mice at the proestrus phase. In this manner, depressive-like behavior is predominant at the proestrus phase of the estrous cycle since the degree of statistical significance is higher btw male vs. proestrus than male vs. estrus.

Like the human follicular stage, the proestrus phase is associated with a rise in circulating estradiol, prolactin, and luteinizing hormone before the release of the follicle-stimulating hormone whose peak is associated with a rapid decline in estradiol levels at ovulation and estrus phase [15, 19]. Hence, in addition to the alterations in behavioral parameters, the level of corticosterone was highest at the proestrus phase as compared to the other groups of female and male mice. A previous study reported sex-related interaction between the depressive-like behavioral phenotype and corticosterone [34]. Unlike the male mice in this previous study, the female mice exhibited a corticosterone-independent depressive-like response. Additional analysis was carried out to detect possible estrous cycle stage-dependent change in [CORT]. However, overall results showed no significant interaction between data sets from the row (proestrus female sham and estrus female sham) and column (proestrus female, estrus female, and male exposed to either FST or TST). A statistically non-significant interaction means that the sham female at proestrus or estrus and behavioral intervention (independent variable) is not different from sex/ hormonal background (proestrus female, estrus female, and male) using [CORT] as a dependent variable. This finding provides data on the sham female mice at proestrus and estrus. The [CORT] of these groups of mice could aid the detection of unsuspecting stress and facilitate the assessment of severity in laboratory animals. Hence, this outcome is applicable to different experimental settings. Healthy responses, severity assessment, and impaired welfare of animals could be a measure of short- or long-term increase in glucocorticoid concentrations [35].

The sex-related differences support further comparative analysis of mice’s response to pharmacological treatments [36]. The treatment with imipramine or fluoxetine (antidepressants) decreased the immobility time and increased the movement time in the FST and TST. Antidepression-like response to imipramine and fluoxetine treatment among the female (at proestrus and estrus) and male mice was not different significantly after TST and FST exposure. However, this finding is inconsistent with the hypothesis that associates the highest antidepressant-like effect in female mice at the proestrus phase marked by depressive-like behavioral phenotype. These findings advance our research proposal with animal models of depression. Different experimental conditions (dimensions, temperature, water depth, test duration, and housing), strains, and age of animals which could interfere with the behavioral endpoint will be explored to expand the study scope and outcome.

## Conclusion

Here, we report sex-related differences to deepen behavioral phenotyping, provide additional insight into the inclusion of male and female subjects in research, and preclinical screening in depression models. The corticosterone level and response to antidepressant drugs in this study provide additional insights that advanced our understanding of sex-oriented response to pharmacological intervention in these models.

## Supporting information

S1 File Supporting data of experiments showing in the figures. (DOCX)

S1 Graphical abstract (TIF)
