# Supplementary material for: A comparative analysis of depressive-like behavior: Exploring sex-related differences and insights
Source: PLoS One. 2023 Nov 29;18(11):e0294904. doi: 10.1371/journal.pone.0294904 (PMC10686438; doi:10.1371/journal.pone.0294904)
Supplement: S1 File — (DOCX) [file pone.0294904.s001.docx]

**Data for figure 1 – 5**

**Data for figure 1**

**A**

| **Female** | **Male** |
| --- | --- |
| 75 | 85 |
| 81 | 90 |
| 71 | 80 |
| 68 | 76 |
| 72 | 92 |
| 74 | 77 |
| 66 | 76 |
| 70 | 92 |
| 74 | 83 |
| 77 | 86 |

**B**

| **Female** | **Male** | **Female** | **Male** |
| --- | --- | --- | --- |
| 27 | 27 | 27.7 | 27.6 |
| 25 | 28 | 24 | 20 |
| 20.1 | 27.7 | 22.5 | 27 |
| 23.5 | 27.2 | 22.2 | 25.8 |
| 29.5 | 28.5 | 30 | 31.5 |
| 27.2 | 24 | 27.2 | 28 |
| 28.5 | 22.5 | 28.5 | 27.7 |
| 24 | 22.2 | 24 | 27.2 |
| 22.5 | 23 | 22.5 | 32 |
| 26 | 25 | 25 | 28 |

**C**

| **Sham** | **SPT** | **FST** | **TST** |
| --- | --- | --- | --- |
| 0.1 | 0.24 | 0.206 | 0.272 |
| 0.124 | 0.209 | 0.141 | 0.212 |
| 0.126 | 0.133 | 0.28 | 0.4 |
| 0.125 | 0.219 | 0.318 | 0.34 |
| 0.23 | 0.34 | 0.2 | 0.222 |
| 0.129 | 0.13 | 0.118 | 0.245 |
| 0.19 | 0.13 | 0.218 | 0.241 |
| 0.116 | 0.1024 | 0.27 | 0.2 |
| 0.131 | 0.139 | 0.3 | 0.12 |
| 0.12 | 0.114 | 0.146 | 0.244 |

**D**

| **Sham** | **SPT** | **FST** | **TST** |
| --- | --- | --- | --- |
| 0.13 | 0.124 | 0.346 | 0.372 |
| 0.224 | 0.209 | 0.2 | 0.212 |
| 0.129 | 0.13 | 0.318 | 0.244 |
| 0.229 | 0.124 | 0.173 | 0.374 |
| 0.116 | 0.139 | 0.335 | 0.463 |
| 0.131 | 0.114 | 0.346 | 0.12 |
| 0.12 | 0.133 | 0.318 | 0.244 |
| 0.126 | 0.219 | 0.2 | 0.222 |
| 0.125 | 0.34 | 0.118 | 0.245 |
| 0.23 | 0.13 | 0.218 | 0.241 |

**Data for Figure 2**

**A**

| **FST** | **TST** |
| --- | --- |
| 90 | 109.62 |
| 90 | 132 |
| 74 | 109.9 |
| 88 | 145 |
| 94 | 161 |
| 94 | 110 |
| 86 | 123 |
| 70 | 112 |
| 100 | 128 |
| 75 | 145 |

**B**

| **FST** | **TST** |
| --- | --- |
| 190 | 110 |
| 140 | 107 |
| 180 | 130 |
| 215 | 100 |
| 235 | 181 |
| 188 | 120 |
| 135 | 99 |
| 194 | 121 |
| 187 | 112 |
| 221 | 198 |

**C**

| **FST** | **TST** |
| --- | --- |
| 145 | 203 |
| 160 | 218 |
| 177 | 194 |
| 165 | 215 |
| 181 | 210 |
| 145 | 203 |
| 160 | 218 |
| 177 | 194 |
| 165 | 215 |
| 181 | 210 |

**D**

| **FST** | **TST** |
| --- | --- |
| 124 | 77 |
| 130 | 100 |
| 116 | 55 |
| 145 | 68 |
| 150 | 74 |
| 124 | 77 |
| 130 | 100 |
| 116 | 55 |
| 145 | 68 |
| 150 | 74 |

**E**

| **FST** | **FST** |
| --- | --- |
| 90 | 145 |
| 90 | 160 |
| 74 | 177 |
| 88 | 165 |
| 94 | 181 |
| 90 | 145 |
| 90 | 160 |
| 74 | 177 |
| 88 | 165 |
| 94 | 181 |

**F**

| **FST** | **FST** |
| --- | --- |
| 190 | 124 |
| 140 | 130 |
| 180 | 116 |
| 215 | 145 |
| 235 | 150 |
| 190 | 124 |
| 140 | 130 |
| 180 | 116 |
| 215 | 145 |
| 235 | 150 |

**G**

| **TST** | **TST** |
| --- | --- |
| 109.62 | 203 |
| 132 | 218 |
| 109.9 | 194 |
| 145 | 215 |
| 161 | 210 |
| 109.62 | 203 |
| 132 | 218 |
| 109.9 | 194 |
| 145 | 215 |
| 161 | 210 |
|  |  |

**H**

| **TST** | **TST** |
| --- | --- |
| 110 | 77 |
| 107 | 100 |
| 130 | 55 |
| 100 | 68 |
| 181 | 74 |
| 110 | 77 |
| 107 | 100 |
| 130 | 55 |
| 100 | 68 |
| 181 | 74 |

**Data for Figure 3**

**A**

| **Proestrus** | **Estrus** | **Male** |
| --- | --- | --- |
| 45 | 112 | 176 |
| 85 | 88 | 144 |
| 95 | 122.2 | 98 |
| 90 | 114 | 156 |
| 88 | 78 | 88 |
| 76 | 76.1 | 147 |
| 90 | 95 | 83 |
| 85.6 | 102 | 135 |
| 87 | 116 | 150 |
| 114,4 | 117 | 172 |

**B**

| **Proestrus** | **Estrus** | **Male** |
| --- | --- | --- |
| 202.3 | 154 | 176 |
| 185 | 188 | 144 |
| 195 | 141 | 102 |
| 195 | 168 | 156 |
| 188 | 178 | 98 |
| 152.4 | 176 | 147 |
| 189 | 195 | 100 |
| 192.9 | 175.6 | 135.6 |
| 204 | 185 | 150 |
| 216.8 | 194 | 141 |

**C**

| **Proestrus** | **Estrus** | **Male** |
| --- | --- | --- |
| 180.3 | 144 | 176 |
| 150 | 170 | 264 |
| 155 | 110 | 198 |
| 125 | 168 | 168 |
| 221.6 | 178 | 188 |
| 152.4 | 176 | 177 |
| 152.9 | 165.6 | 205.6 |
| 154 | 164 | 238 |
| 98 | 185 | 218 |
| 137 | 194 | 222 |

**D**

| **Proestrus** | **Estrus** | **Male** |
| --- | --- | --- |
| 178.3 | 144 | 176 |
| 142.9 | 125.6 | 85.6 |
| 150.3 | 110 | 264 |
| 154 | 110 | 198 |
| 125 | 151 | 168 |
| 131.6 | 147 | 188 |
| 151.4 | 100 | 177 |
| 153 | 124 | 238 |
| 102 | 115 | 218 |
| 139 | 124 | 222 |

**Data for figure 4**

**A**

| **Sham Female** | **Female at Proestrus** | **Female at Estrus** | **Sham Male** | **Male** |
| --- | --- | --- | --- | --- |
| 0.141 | 0.246 | 0.172 | 0.141 | 0.172 |
| 0.19 | 0.181 | 0.212 | 0.09 | 0.212 |
| 0.141 | 0.229 | 0.24 | 0.141 | 0.24 |
| 0.029 | 0.303 | 0.244 | 0.029 | 0.244 |
| 0.1 | 0.218 | 0.274 | 0.1 | 0.174 |

**B**

| **Sham Female** | **Female at Proestrus** | **Female at Estrus** | **Sham Male** | **Male** |
| --- | --- | --- | --- | --- |
| 0.141 | 0346 | 0.372 | 0.141 | 0.22 |
| 0.129 | 0.371 | 0.212 | 0.129 | 0.212 |
| 0.141 | 0.29 | 0.24 | 0.141 | 0.24 |
| 0.029 | 0.43 | 0.244 | 0.029 | 0.244 |
| 0.1 | 0.318 | 0.374 | 0.1 | 0.274 |

|  | **Proestrus Female** | | | | | **Estrus Female** | | | | | **Male** | | | | |
| --- | --- | --- | --- | --- | --- | --- | --- | --- | --- | --- | --- | --- | --- | --- | --- |
| **Proestrus Female Sham** | 0.172 | 0.212 | 0.26 | 0.2 | 0,16 | 0.206 | 0.121 | 0.109 | 0.29 | 0.218 | 0.132 | 0.08 | 0.18 | 0.14 | 0.174 |
| **Estrus Female Sham** | 0.181 | 0.209 | 0.15 | 0.18 | 0.24 | 0.141 | 0.19 | 0.161 | 0.03 | 0.14 | 0.13 | 0.12 | 0.141 | 0.026 | 0.14 |

|  | **Proestrus Female** | | | | | **Estrus Female** | | | | | **Male** | | | | |
| --- | --- | --- | --- | --- | --- | --- | --- | --- | --- | --- | --- | --- | --- | --- | --- |
| **Proestrus Female Sham** | 0.172 | 0.212 | 0.26 | 0.2 | 0,16 | 0.206 | 0.121 | 0.109 | 0.29 | 0.218 | 0.132 | 0.08 | 0.18 | 0.14 | 0.174 |
| **Estrus Female Sham** | 0.181 | 0.209 | 0.15 | 0.18 | 0.24 | 0.141 | 0.19 | 0.161 | 0.03 | 0.14 | 0.13 | 0.12 | 0.141 | 0.026 | 0.14 |

**C**

|  | **Proestrus Female** | | | | | **Estrus Female** | | | | | **Male** | | | | |
| --- | --- | --- | --- | --- | --- | --- | --- | --- | --- | --- | --- | --- | --- | --- | --- |
| **Proestrus Female Sham** | 0.172 | 0.212 | 0.26 | 0.2 | 0,16 | 0.206 | 0.121 | 0.109 | 0.29 | 0.218 | 0.132 | 0.08 | 0.18 | 0.14 | 0.174 |
| **Estrus Female Sham** | 0.181 | 0.209 | 0.15 | 0.18 | 0.24 | 0.141 | 0.19 | 0.161 | 0.03 | 0.14 | 0.13 | 0.12 | 0.141 | 0.026 | 0.14 |

**D**

|  | **Proestrus Female** | | | | | **Estrus Female** | | | | | **Male** | | | | | |
| --- | --- | --- | --- | --- | --- | --- | --- | --- | --- | --- | --- | --- | --- | --- | --- | --- |
| **Proestrus Female Sham** | 0.346 | 0.212 | 0.371 | 0.29 | 0.43 | 0.206 | 0.374 | 0.372 | 0.244 | 0.218 | 0.15 | 0.108 | 0.18 | 0.18 | 0.274 |  |
| **Estrus Female Sham** | 0.318 | 0.209 | 0.15 | 0.18 | 0.24 | 0.141 | 0.24 | 0.19 | 0.3 | 0.14 | 0.129 | 0.141 | 0.141 | 0.2 | 0.18 |  |

**Data for figure 5 (**P: proestrus; E: estrus; M: male**)**

**A**

| **E Vehicle** | **M Vehicle** | **P IMI 15 mg/Kg** | **E IMI 15 mg/Kg** | **M IMI 15 mg/Kg** | **P FLX 15 mg/kg** | **E FLX 15 mg/kg** | **M FLX 15 mg/kg** |
| --- | --- | --- | --- | --- | --- | --- | --- |
| 193 | 200.8 | 176 | 170 | 165 | 140 | 136 | 122 |
| 153 | 205 | 178.8 | 165 | 151 | 141 | 134 | 94.8 |
| 206 | 182,9 | 230 | 175 | 149 | 135 | 138 | 118 |
| 215 | 224 | 156 | 181 | 181 | 128.9 | 140 | 135 |
| 201 | 201 | 187 | 158 | 158 | 181.7 | 118 | 141.5 |
| 200 | 179.5 | 157 | 181 | 163.8 | 110.4 | 122 | 121 |
| 158 | 227 | 159 | 131.5 | 187 | 132.7 | 151 | 118 |
| 214.6 | 190 | 164 | 172 | 146 | 142 | 122 | 132 |
| 198 | 189 | 166 | 205.8 | 166 | 130 | 142 | 120 |
| 194 | 200.5 | 186 | 161 | 183 | 158 | 158 | 118 |

**B**

| **P Vehicle** | **E Vehicle** | **M Vehicle** | **P IMI 15 mg/Kg** | **E IMI 15 mg/Kg** | **M IMI 15 mg/Kg** | **P FLX 15 mg/kg** | **E FLX 15 mg/kg** | **M FLX 15 mg/kg** |
| --- | --- | --- | --- | --- | --- | --- | --- | --- |
| 134 | 150 | 147 | 173 | 180 | 170 | 180 | 192 | 196 |
| 139 | 155 | 119 | 179 | 202 | 158 | 186 | 201 | 216.5 |
| 118.8 | 160 | 120 | 160 | 140.2 | 169 | 151,5 | 210 | 199 |
| 141 | 140 | 157 | 180 | 165.2 | 198 | 156 | 189 | 176.8 |
| 168.9 | 183.5 | 166 | 190 | 169 | 208 | 175 | 163 | 212.5 |
| 108 | 110 | 151 | 181 | 202.9 | 159 | 190 | 212 | 212 |
| 128 | 163 | 140 | 143.5 | 188 | 150 | 196 | 186 | 185 |
| 130 | 112 | 144 | 197.2 | 180 | 155 | 200 | 163.5 | 180 |
| 139 | 158 | 159 | 165 | 196.4 | 158 | 187 | 185 | 198 |
| 133 | 168 | 169 | 161 | 176 | 175 | 178 | 218.5 | 184 |

**C**

| **P Vehicle** | **E Vehicle** | **M Vehicle** | **P IMI 15 mg/Kg** | **E IMI 15 mg/Kg** | **M IMI 15 mg/Kg** | **P FLX 15 mg/kg** | **E FLX 15 mg/kg** | **M FLX 15 mg/kg** |
| --- | --- | --- | --- | --- | --- | --- | --- | --- |
| 190.6 | 180.6 | 171 | 150 | 145 | 133 | 119 | 114 | 100 |
| 191 | 186 | 156 | 137 | 122.5 | 145 | 110 | 123 | 61 |
| 185 | 165 | 177 | 154 | 154 | 128 | 148 | 90 | 90 |
| 200 | 165.9 | 196 | 166 | 165.4 | 90 | 115 | 138.9 | 135 |
| 176 | 170 | 170 | 165 | 145 | 118 | 116 | 121 | 121 |
| 152.2 | 201 | 181 | 145 | 160 | 147 | 134 | 107 | 85 |
| 227 | 212 | 189 | 145 | 132.2 | 162 | 139 | 110 | 117 |
| 188 | 154 | 170 | 162 | 136 | 159 | 84 | 88.6 | 76 |
| 190 | 178 | 176 | 170.8 | 125.2 | 125 | 99 | 119 | 109 |
| 201 | 188 | 124 | 105 | 165 | 123 | 126 | 128 | 106 |

**D**

| **P Vehicle** | **E Vehicle** | **M Vehicle** | **P IMI 15 mg/Kg** | **E IMI 15 mg/Kg** | **M IMI 15 mg/Kg** | **P FLX 15 mg/kg** | **E FLX 15 mg/kg** | **M FLX 15 mg/kg** |
| --- | --- | --- | --- | --- | --- | --- | --- | --- |
| 140.6 | 155.6 | 150 | 170 | 195 | 192 | 200 | 210 | 220 |
| 152 | 144 | 138 | 176 | 198 | 200.8 | 175.5 | 218 | 235 |
| 165 | 150 | 158 | 190 | 194 | 206.2 | 185 | 190.2 | 232 |
| 108 | 146 | 150 | 156 | 213.5 | 176.9 | 225 | 175 | 249 |
| 135 | 152 | 166 | 153 | 205 | 200 | 201 | 214 | 245 |
| 146 | 150 | 170 | 208.8 | 176 | 142.6 | 202 | 210 | 238 |
| 150 | 185 | 160 | 163 | 214.2 | 188 | 186 | 211 | 159.5 |
| 140 | 141 | 156 | 157 | 210 | 220.8 | 210 | 238 | 227 |
| 138 | 146 | 115.8 | 172 | 168 | 197 | 189 | 246 | 240 |
| 142 | 165 | 160 | 158 | 157 | 187 | 238 | 198 | 225 |
| 130 | 170 | 126 | 166 | 214.6 | 200.5 | 188 | 200 | 149.9 |
