# Supplementary figures and images for: A comparative analysis of depressive-like behavior: Exploring sex-related differences and insights
Source: PLoS One. 2023 Nov 29;18(11):e0294904. doi: 10.1371/journal.pone.0294904 (PMC10686438; doi:10.1371/journal.pone.0294904)

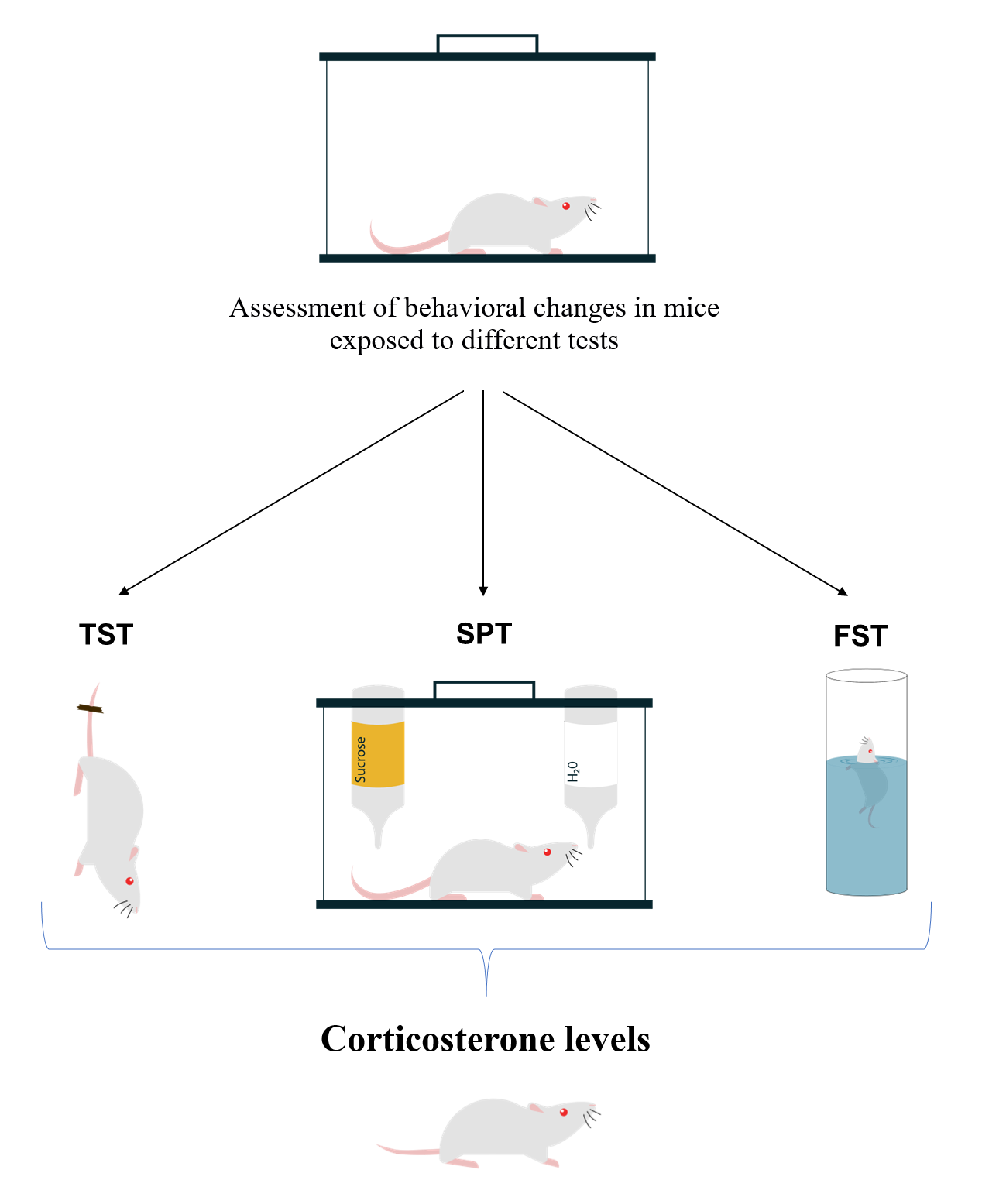

Supplement: S1 Graphical abstract — (TIF) [file pone.0294904.s002.tif]
